# Genetic polymorphisms in plasminogen activator inhibitor-1 predict susceptibility to steroid-induced osteonecrosis of the femoral head in Chinese population

**DOI:** 10.1186/1746-1596-8-169

**Published:** 2013-10-17

**Authors:** Yanqiong Zhang, Rongtian Wang, Shangzhu Li, Xiangying Kong, Zhiyao Wang, Weiheng Chen, Na Lin

**Affiliations:** 1 Institute of Chinese Materia Medica, China Academy of Chinese Medical Sciences, No. 16, Nanxiaojie, Dongzhimennei, Beijing 100700, China; 2 Wang Jing Hospital (Hospital of Orthopedics and Traumatology), China Academy of Chinese Medical Sciences, Beijing 100102, China; 3 Institute of Hematology & Blood Diseases Hospital, Chinese Academy of Medical Sciences & Peking Union Medical College, Tianjin 300041, China

**Keywords:** Steroid-induced osteonecrosis of the femoral head, Plasminogen activator inhibitor-1, Single nucleotide polymorphism

## Abstract

**Background:**

Steroid usage has been considered as a leading cause of non-traumatic osteonecrosis of the femoral head (ONFH), which is involved in hypo-fibrinolysis and blood supply interruption. Genetic polymorphisms in plasminogen activator inhibitor-1 (PAI-1) have been demonstrated to be associated with ONFH risk in several populations. However, this relationship has not been established in Chinese population. The aim of this study was to investigate the association of PAI-1 gene polymorphisms with steroid-induced ONFH in a large cohort of Chinese population.

**Methods:**

A case–control study was conducted, which included 94 and 106 unrelated patients after steroid administration recruited from 14 provinces in China, respectively. Two SNPs (rs11178 and rs2227631) within PAI-1 were genotyped using Sequenom MassARRAY system.

**Results:**

rs2227631 SNP was significantly associated with steroid-induced ONFH group in codominant (P = 0.04) and recessive (P = 0.02) models. However, there were no differences found in genotype frequencies of rs11178 SNP between controls and patients with steroid-induced ONFH (all P > 0.05).

**Conclusions:**

Our data offer the convincing evidence for the first time that rs2227631 SNP of PAI-1 may be associated with the risk of steroid-induced ONFH, suggesting that the genetic variations of this gene may play an important role in the disease development.

**Virtual slides:**

The virtual slide(s) for this article can be found here: http://www.diagnosticpathology.diagnomx.eu/vs/1569909986109783.

## Introduction

Non-traumatic osteonecrosis of femoral head (ONFH) represents an intractable bone disease, pathophysiologically characterized by progressive collapse of the femoral head because of a disturbance in the supply of blood and anomalies in the fibrinolytic system [1,2]. Since this ischemic necrosis of the femoral head and deterioration of hip joint function may significantly affect patient quality of life, it is urgent to clarify the pathogenic mechanisms regarding this disease. Recent studies have demonstrated that various factors, such as steroid usage, alcoholism, infections, marrow infiltrating diseases, coagulation defects and some autoimmune diseases may be associated with non-traumatic ONFH [3-5]. Among them, steroid usage has been considered as a leading cause of non-traumatic ONFH. Currently, more than 30 million Americans require steroid drugs as a part of their treatment regime, and up to 40% of steroid users develop some degree of ONFH depending on both the duration of therapy and dose [6]. There are three pathophysiological characteristics on patients with steroid-induced ONFH as following: (1) Steroid-induced ONFH is an iatrogenic disease and develops at a very early stage during steroid administration; (2) Most patients with symptomatic steroid-induced ONFH eventually need surgery (usually total hip arthroplasty) within a few years of onset, however, these patients often require multiple increasingly difficult surgeries over the course of a lifetime because the average age at presentation is very young (about 33 years of age); (3) Not all patients with steroid administration develop steroid-induced ONFH, which may be caused by the individual variation to steroids sensitivity [7,8]. On the basis of above characteristics, it is of great significance to identify novel and useful risk factors to predict which patients receiving a specific dose of steroid will develop ONFH, to indicate individual differences in steroid sensitivity and to investigate the potential of additional pathogenic mechanisms.

Although the pathogenic mechanisms of steroid-induced ONFH have not been fully elucidated, recent studies have demonstrated that the development of this disease may be associated with blood coagulation in the femoral head, which is mainly caused by the damage of the endotheliocyte membrane, as well as the tendency for blood to congeal [9,10]. Plasminogen activator inhibitor (PAI)-1 is one of the most important regulators for the balance of the coagulation and fibrinolytic systems [11]. It is the primary inhibitor of both tissue- and urinary-type plasminogen activators. Changes in PAI-1 may lead to a destabilization of the fibrinolytic system directly, destroying the balance between blood coagulation and fibrinolysis [12]. The product of coagulation accumulates in vessels easily, and thus, the blood stream will be limited or disrupted completely [13]. Accumulating evidences have suggested the relationship between abnormalities of PAI-1 and ONFH development. Tan et al. [14] showed that the expression level of PAI-1 protein was significantly upregulated in the sera of patients with idiopathic ONFH detected by enzyme-linked immunosorbent assay (ELISA); Hozumi et al. [15] reported that PAI-1 expression at both mRNA and protein levels was significantly increased by steroid adiministration and bone marrow adipocytes may play important roles in the development of steroid-induced ONFH by enhancing PAI-1 expression. They also found that simvastatin may exhibit preventive effects against steroid-induced ONFH by suppressing PAI-1 secretion [16]. PAI-1 antigen levels are determined by genetic polymorphisms within the PAI-1 gene. It has been indicated that the plasma PAI-1 antigen level may be higher in the 4G/4G genotype than in the 5G/5G genotype [17]; Glueck et al. [18] first reported that the genotype frequency of 4G/4G was 41% in ONFH patients and 20% in healthy control subjects from USA; Ferrari et al. [19] investigated the relationship between the incidence of postrenal transplant ONFH and PAI-1 4G/5G polymorphism in Swiss population and reported that development of ONFH may be more frequently observed in the 4G/4G genotype; Kim et al. [20] also found that the 4G allele of rs1799889, A allele of rs2227631, and C allele of rs11178 were significantly associated with increased ONFH risk in Korea population. In contrast, Asano et al. [21] using Japanese population suggested that plasma PAI-1 levels were highest in ONFH patients with the 4G/4G genotype, but that the incidence of ONFH was not related to this genotype. These findings indicated that the genetic polymorphisms within the PAI-1 gene may play an important role in the development of ONFH.

Since the relationship between the genetic polymorphisms of the PAI-1 gene and the risk of steroid-induced ONFH has not been determined in Chinese population, we performed the current study to better understand the association of PAI-1 gene polymorphisms with steroid-induced ONFH in a large cohort of Chinese population.

## Materials and methods

### Study subjects

The study was approved by the Research Ethics Committee of Institute of Chinese Materia Medica, Wangjing Hospital, & Institute of Hematology & Blood Diseases Hospital, China Academy of Chinese Medical Sciences, China. Informed consent was obtained from all of the patients.

A total of 94 patients with steroid-induced ONFH (case group, 40 men, 54 women; mean age: 40.22 ± 14.78 years) and 106 patients who did not develop steroid-induced ONFH (reference group, 64 men, 42 women; mean age: 43.09 ± 18.36 years) following steroid administration were consecutively enrolled at Wangjing Hospital and Institute of Hematology & Blood Diseases Hospital of China Academy of Chinese Medical Sciences from March 2011 to December 2012. All the subjects are from 14 provinces in China, including Hebei (n = 41), Beijing (n = 32), Tianjin (n = 27), Shandong (n = 21), Henan (n = 18), Liaoning (n = 14), Jilin (n = 11), Shanxi (n = 10), Heilongjiang (n = 10), Neimenggu (n = 8), Guizhou (n = 3), Ningxia (n = 2), Shanxi (n = 2) and Qinghai (n = 1). Steroid-induced ONFH was defined by a history of a mean daily dose of ≥16.6 mg or highest daily dose of 80 mg of predinosolone equivalent within 1 year prior to the development of symptoms or radiological diagnosis in asymptomatic cases [22-24]. Underlying diseases in steroid-induced ONFH were hematologic diseases (n = 23 patients), dermatogic diseases (n = 9 patients), renal diseases (n = 9 patients), ophthalmopathy (n = 6 patients), diseases of respiratory system (n = 5 patients), and others (n = 42 patients). Patients with a demonstrable history of direct trauma or with possible combined causes were excluded. The clinical characteristics of patients in case and reference groups were summarized in Table 1.

**Table 1:** Clinical information of study subjects

**Factor**	**Steroid-induced ONFH (+) Cases (N = 94)**	**Steroid-induced ONFH (-) Cases (N = 106)**	***P***
**Gender**			
Male	40	64	**0.02**
Female	54	42	
**Age (year)**			
Median (Range)	38.00 (18–82)	44.50 (18–80)	0.23
Mean	40.22 ± 14.78	43.09 ± 18.36	
**Body mass index (BMI, Kg/m**^**2**^**)**			
Median (Range)	22.8 (16.9-30.8)	24.45 (18.48-30.12)	**<0.001**
Mean	23.01 ± 3.00	24.36 ± 2.42	
**Concurrent or past medical history**			
Hematologic diseases	23	106	**<0.001**
Dermatogic diseases	9	0	
Renal diseases	9	0	
Ophthalmopathy	6	0	
Diseases of respiratory system	5	0	
Others	42	0	
**Affected**			
Unilateral	15	**-**	**-**
Bilateral	79	**-**	
**Steroid treatment**			
Mouth medication	74	89	0.06
Intravenous injection	36	71	
**Steroid dose**			
Large (>400 mg/d)	34	60	0.12
Middle (>100 mg/d & < 400 mg/d)	52	40	
Small	8	6	
**Days of steroid treatment**			
Median (Range)	360 (7–9490)	810 (4–79934)	0.10
Mean	1140.34 ± 2014.037	2631.54 ± 8610.278	

Note: "Bolded values" refer to the statistically significant data.

### Candidate PAI-1 single nucleotide polymorphism (SNP) selection

Two well-studied functional SNPs in the PAI-1 gene, rs2227631 (-844 G/A, in the promoter) and rs11178 (+10700 C/T, in the 3′UTR), were selected in the list of genotyping in this study for their important functions in regulating PAI-1 expression and in the development of ONFH. As the results of our literature retrieval from PubMed database ( http://www.ncbi.nlm.nih.gov/sites/entrez?db=PubMed), Kim et al. [20] showed for the first time that rs2227631 and rs11178 are essential SNPs involved in the regulation of PAI-1 gene expression in ONFH using a Korean population. According to the subgroup analysis, they confirmed the risk effects of rs2227631 and rs11178 in the idiopathic ONFH and men subgroups, and those of rs11178 in the alcohol-induced ONFH subgroup. However, no association was seen in the steroid-induced ONFH group because of the small sample size. In the current study, the relationships between rs2227631 and rs11178 SNPs, and the development of steroid-induced ONFH were investigated using a large cohort of Chinese population.

### DNA isolation

Genomic DNA was extracted from 2 mL whole-blood samples using the QIAamp DNA Blood Mini kit (Qiagen, Inc., Valencia, CA) following the manufacturer’s protocol. After dilution to 20 ng/μL, DNA was distributed in 96-well plates and stored at -80°C.

### PAI-1 SNP genotyping

**Figure 1:**
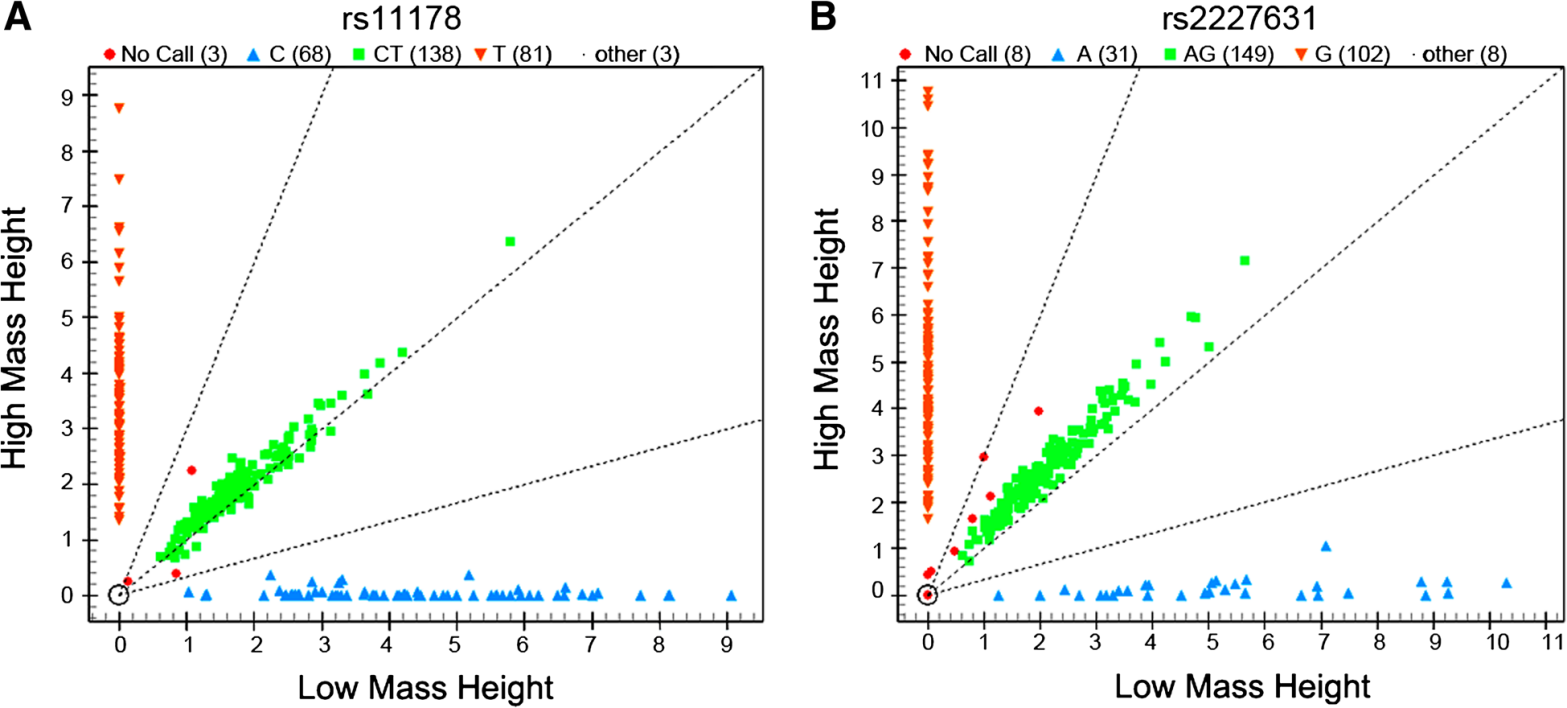
**Clustering graphs of genotypes for rs11178 (+10700 C/T in the 3′UTR, A) and rs2227631 (-844 G/A in the promoter, B).** The genotyping success rate was over 95%.

SNP genotyping was performed on the SEQUENOM MassARRAY® Analyzer 4 (Sequenom, Inc., San Diego, CA, USA) using genomic DNA in a single multiplex reaction. Primers for polymerase chain reaction (PCR) amplification and single base extension were designed by Sequenom Assay Design 3.1 software (Sequenom, San Diego, CA, USA) according to the manufacturer’s instructions (Table 2). For quality control, genotyping was performed without knowledge of the case/control status of the subjects, and a random sample of 5% of cases and controls was genotyped again by different researchers. The reproducibility was 100%. The genotyping success rate was over 95% (Figure 1).

**Table 2:** Polymerase chain reaction primers of selected SNPs

**Single nucleotide polymorphism**	**PCR primer**	**Sequence**	**Extension primer**	**Sequence**
rs11178	1^st^-primer	ACGTTGGATGATAGTTTCTACCAGGCACAC	1^st^-primer	AGGCCCTTTGCAGGAC
	2^nd^-primer	ACGTTGGATGAGACCTGGTTCCCACTGAG	2^nd^-primer	AGGCCCTTTGCAGGAT
rs2227631	1^st^-primer	ACGTTGGATGAAGGAAACAGGAGACCAACG	1^st^-primer	gcaTAGCGGGCAGCTCGAA
	2^nd^-primer	ACGTTGGATGAGGATAAAGGACAAGCTGCC	2^nd^-primer	gcaTAGCGGGCAGCTCGAG

### Statistical analysis

The software of SPSS version12.0 for Windows (SPSS Inc, IL, USA) and SAS 9.1 (SAS Institute, Cary, NC) was used for statistical analysis. Continuous variables were expressed as $\overset{¯}{X}\pm s$. Statistical analysis were performed with Fisher's exact test for any 2 × 2 tables,Pearson χ^2^ test for non- 2 × 2 tables, chi-square trend test for ordinal datum. A significant departure of genotype frequency from Hardy-Weinberg equilibrium (HWE) for each SNP was estimated using SNPStats ( http://bioinfo.iconcologia.net/snpstats/start.htm). Significant differences in genotype and allele frequency between steroid-induced ONFH cases and controls were assessed using the χ^2^ test for categorical variables. An association analysis based on unconditional binary logistic regression was carried out to determine the odds ratio (OR) and 95% confidence interval (95% CI) for each SNP, using age, gender, body mass index (BMI), concurrent or past medical history, steroid dose and days of steroid treatment as covariates. The level of significance was set at P < 0.05.

## Results

### Participant demographics

Table 1 shows the general characteristics of the study population which consisted of 94 steroid-induced ONFH patients (case group) and 106 patients who received steroid administration but did not develop steroid-induced ONFH (reference group). The subjects in the reference group were well matched with those in the case group for age, steroid treatment, steroid dose, and days of steroid treatment (all P > 0.05, Table 1). However, there were significant differences in the gender (P = 0.02), BMI (P < 0.001), and past medical history (P < 0.001) distributions between the two groups. The steroid-induced ONFH patients were more likely in men subgroup, and to have a smaller BMI when compared to the controls. In order to avoid the influence of these mismatch factors on our statistical results, we used them as covariates in logistic regression analysis.

#### rs2227631 associates with the risk for steroid-induced ONFH

The frequencies of rs11178 and rs2227631 SNPs were tested in Hardy-Weinberg formula by SNPStats ( http://bioinfo.iconcologia.net/snpstats/start.htm). The results showed that all genotype distributions of the case and reference groups were coherent with the assumption of Hardy-Weinberg equilibrium [(HWE), rs11178 (P = 0.47) and rs2227631 (P = 0.28)] (P > 0.05).

We further found significant associations of genotypic and allelic frequencies for rs11178 and rs2227631 SNPs of PAI-1 gene with steroid-induced ONFH. As shown in Table 3, rs2227631 SNP was significantly associated with steroid-induced ONFH group in codominant (P = 0.04) and recessive (P = 0.03) models. However, there were no differences found in genotype frequencies of rs11178 SNP between controls and patients with steroid-induced ONFH (all P > 0.05, Table 3).

**Table 3:** Association between rs11178 and rs2227631 SNPs and the risk of steroid-induced ONFH

**SNP ID**	**Genotype**	**Cases**	**Control**	**Codominant**		**Dominant**		**Recessive**		**Overdominant**	
				**OR (95% CI)**	**P**	**OR (95% CI)**	**P**	**OR (95% CI)**	**P**	**OR (95% CI)**	**P**
rs11178	C/C	21	24	1.00	0.15	1.00	0.07	1.00	0.23	1.00	0.55
	T/C	52	52	0.50		0.39		0.66		0.80	
				(0.20-1.23)		(0.16- 1.08)		(0.28-1.47)		(0.39-1.65)	
	T/T	20	27	0.38		0.46		0.60		0.81	
				(0.13-1.07)		(0.19-1.07)		(0.26-1.40)		(0.39-1.68)	
rs2227631	G/G	34	33	1.00	**0.04**	1.00	0.18	1.00	**0.02**	1.00	0.83
	A/G	43	53	0.74		0.60		0.24		1.08	
				(0.34-1.63)		(0.28-1.27)		(0.07-0.80)		(0.53-2.20)	
	A/A	9	14	0.20		0.53		0.22		1.05	
				(0.05-0.73)		(0.30-0.94)		(0.06-0.77)		(0.47-2.12)	

Note: "Bolded values" refer to the statistically significant data.

## Discussion

Steroid exhibits diverse activities in multiple organs and hypercortisolism may cause various disorders including ONFH which is a common complication induced by high-dose administration of steroid [25]. The disease pathogenesis seems to be multifactorial and is still unclear. Recent studies have demonstrated that both hypo-fibrinolysis and corresponding blood supply interruption are major features in the development of steroid-induced ONFH [26]. The genetic variations of genes which are implicated in these pathophysiological processes have been hypothesized to be associated with steroid-induced ONFH. In the present study, we determined the contributions of PAI-1 gene SNPs to steroid-induced ONFH. We demonstrated for the first time that there may be a significant association between rs2227631 SNP (-844 G/A) of the PAI-1 gene and steroid-induced ONFH in Chinese population and the A allele considerably increased disease risk.

It is well known that the damage of the endotheliocyte membrane leading to the tendency for blood to congeal is the key cause of blood coagulation [27]. PAIs may play important roles in this process. There are two forms of PAIs, PAI-1 and PAI-2. PAI-1 is a serine protease inhibitor which is synthesized and released by endothelial cells in blood vessel walls and PAI-2 is a thromboblastic product [28]. PAI-1 exerts its regulatory activity on fibrinolysis by forming complexes with tissue plasminogen activator (t-PA) which may activate plasminogen. Changes in t-PA and PAI-1 lead to a destabilization of the fibrinolytic system directly, destroying the balance between blood coagulation and fibrinolysis [29]. Since blood coagulation in the femoral head is thought to be associated with ONFH, there have been several reports regarding increased PAI-1 secretion in blood sera of patients with ONFH. The human PAI-1 gene, also known as the SERPINE1 gene, is mapped on chromosome 7q21.3-q22, and several polymorphisms within this gene have been described [30]. Among them, 4G/5G SNP may contribute to the strongest SNP association with plasma PAI-1 concentration. High PAI-1 expression levels, induced by -675 4G/5G SNP in the PAI-1 promoter, lead to suppression of fibrinolysis through inhibition of plasminogen activator and promotion of thrombosis [17]. The resulting increase in intraosseous venous pressure which restricts flow to the femoral head may culminate in osteonecrosis [18]. Previous study has reported that the frequency of the 4G/4G genotype was greater in the patients with ONFH after renal transplantation than in the patients without ONFH, suggesting the association between 4G/5G SNP and the development of ONFH in Swiss population [19]. On the contrary, in Japanese population, it has not been demonstrated a statistically significant relationship between the 4G/5G polymorphism and the incidence of ONFH, although the increase in the ONFH development was observed in patients with the PAI-1 4G/4G genotype [21]. Regarding to rs2227631, it is in tight linkage disequilibrium with the widely reported 4G/5G polymorphism in the promoter of the PAI-1 gene, and is potentially implicated in PAI-1 gene regulation [31]. Su et al. [32] indicated that rs2227631 polymorphism was significantly associated with coronary heart disease (CHD) in nonsmokers; in Korean population, this SNP was demonstrated to be a risk factor for ONFH [20], which was in line with the results of our study that confirmed a significant association between rs2227631 polymorphism and the development of steroid-induced ONFH in Chinese population. In addition to rs2227631, rs11178 also significantly associates with increased CHD risk in nonsmokers [32], which might be attributable to strong linkage linkage disequilibrium of this SNP with rs2227631 and rs1799889. For ONFH, recent study of Korean population also identified an association between rs11178 and disease risk [30]. However, our study did not found this relationship in Chinese population. The discrepancy between our results of Chinese population and those of Korean population might be ascribed to the difference in genetic background according to race, which could affect the pathology of steroid-induced ONFH.

There are several advantages in this study as following: (1) we enrolled 94 patients with steroid-induced ONFH in the case group. To the best of our knowledge, the sample size is the largest in genetic polymorphism studies concerning steroid-induced ONFH conducted so far. (2) We compared the polymorphisms between patients having and not having steroid-induced ONFH. These patients had the similar background of steroid treatment, steroid dose, and Days of steroid treatment (without significant differences between case and reference groups), which could not affect the distributions of genetic polymorphisms.

Despite these advantages, there are also several limitations in this study as following: (1) the heterogeneity among patients is a real issue in clinical practice when we are confront with steroid-induced ONFH; (2) the precise mechanisms by which the PAI-1 polymorphisms affect the susceptibility to steroid-induced ONFH are still unclear. Further studies are necessary to fully address this problem.

In conclusion, our data offer the convinced evidence for the first time that rs2227631 SNP of PAI-1 may be associated with the risk of steroid-induced ONFH, suggesting that the genetic variations of this gene may play an important role in the disease development. Taken together, it is helpful to analyze rs2227631 SNP of PAI-1 for identifying susceptibility to steroid-induced ONFH.

## Competing interests

The authors declare that they have no competing interests.

## Authors’ contributions

LN, CW and ZY participated in designing the experiments, writing the paper and revising the manuscript; ZY, WR, LS, KX and WZ carried out the experiments and analyzed data. All authors read and approved the final manuscript.
